# Brachial Artery Aneurysm After Fistula Ligation in a Hemodialysis Patient: A Case Report

**DOI:** 10.7759/cureus.88852

**Published:** 2025-07-27

**Authors:** Mohamed Ouhmich, Youssef Banana, Oussama Anane, Abdellah Rezziki, Adnane Benzirar, Omar El Mahi

**Affiliations:** 1 Department of Vascular Surgery, Mohammed VI University Hospital, Oujda, MAR; 2 Department of Vascular Surgery, Faculty of Medicine and Pharmacy, Mohammed First University, Oujda, MAR

**Keywords:** aneurysm, brachial, fistula, hemodialysis, ligation

## Abstract

Brachial artery aneurysm following arteriovenous fistula (AVF) ligation is an uncommon complication in hemodialysis patients. We report the case of a 53-year-old female patient with a history of humerocephalic AVF ligation who presented with a pulsatile mass in the left elbow crease, absent distal pulses, but no ischemic signs. Surgical resection of the aneurysm and end-to-end brachial artery anastomosis were successfully performed, with good postoperative recovery. This case highlights the importance of clinical vigilance and timely surgical management in patients with prior vascular access surgery. Given the rarity of this condition and the potential for serious complications, clinicians must maintain a high index of suspicion and intervene promptly.

## Introduction

Brachial artery aneurysm following fistula ligation remains uncommon in hemodialysis patients. The reported incidence is around 5-8%, and the etiology remains unknown [1]. These aneurysms can occur after arteriovenous fistula (AVF) ligation, a procedure commonly performed in patients who have received a renal transplant or transitioned off dialysis. Although the pathophysiological mechanisms are not fully elucidated, several contributing factors have been proposed, such as chronic endothelial damage from repeated needling, altered flow dynamics, medial degeneration, and vascular calcification associated with end-stage renal disease.

Patients with brachial artery aneurysms are often symptomatic, typically presenting with a palpable mass, pain, paresthesia, or signs of acute limb ischemia resulting from thromboembolic events. Lesions that are initially asymptomatic become symptomatic in approximately one-third of cases [2].

We report the case of a 53-year-old female patient who underwent humerocephalic fistula ligation one year ago and was admitted with a brachial artery aneurysm, presenting with non-palpable radial and ulnar pulses.

Our case report was written according to the Surgical CAse REport (SCARE) guidelines [3], ensuring the application of structured and transparent surgical case reporting standards.

## Case presentation

We report the case of a 53-year-old woman with end-stage renal disease secondary to hypertensive nephropathy, who had undergone regular hemodialysis via a left humerocephalic AVF for five years. One year prior to admission, she received a successful kidney transplant and underwent surgical ligation of the AVF.

**Figure 1:**
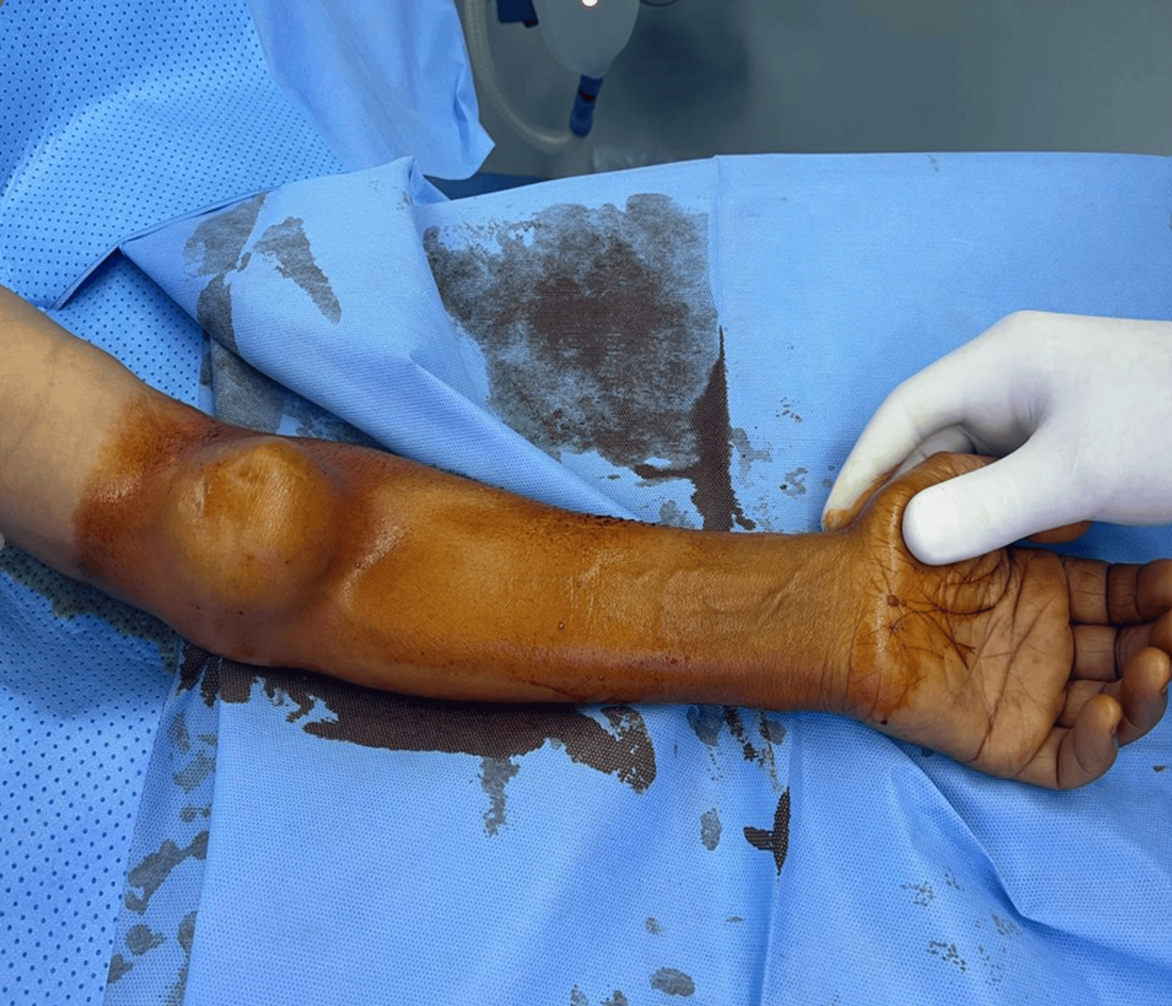
Image showing an aneurysm of the brachial artery at the left elbow crease

The patient presented to our vascular surgery unit with a gradually enlarging, pulsatile mass in the left elbow crease, associated with intermittent discomfort but no sensory or motor deficits. On examination, a well-demarcated, pulsatile, and non-tender mass measuring approximately 7 cm × 5 cm was palpable in the antecubital fossa (Figure 1). Radial and ulnar pulses were absent on palpation, although the hand remained warm and perfused, with no signs of ischemia or neurological impairment.

Doppler ultrasonography and contrast-enhanced CT angiography confirmed a true saccular aneurysm of the brachial artery just proximal to its bifurcation, without signs of thrombosis or distal embolization.

**Figure 2:**
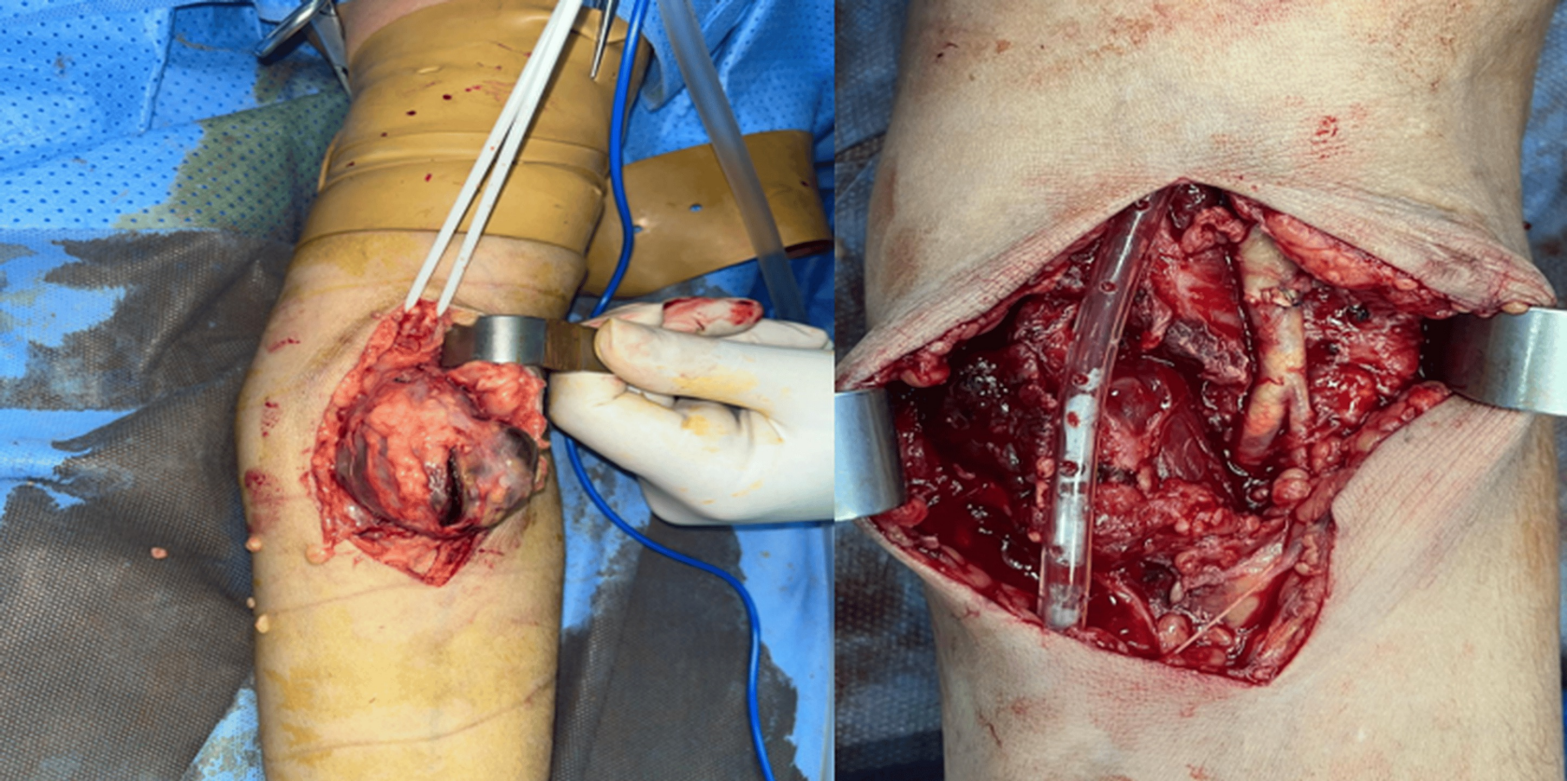
After flattening the aneurysm, an end-to-end anastomosis of the brachial artery was performed just before its bifurcation

Surgical repair was performed under axillary block anesthesia. Intraoperatively, a true aneurysm of the brachial artery was identified and completely resected. A primary end-to-end anastomosis was achieved using 6-0 polypropylene sutures (Figure 2). Postoperative examination confirmed the return of distal pulses, and the patient was discharged on postoperative day 3 with no complications. Follow-up at one and three months revealed no recurrence, preserved distal circulation, and complete functional recovery of the left arm.

## Discussion

The etiology of brachial artery aneurysm development subsequent to AVF ligation remains ambiguous. Teixeira et al. [4] propose a theory involving the upregulation of vasodilator agents, degradation of elastic fibers, and accumulation of calcium and phosphate resulting from dialysis, along with the activation of proinflammatory pathways in immunosuppressed states and long-term corticosteroid therapy, as well as heightened vessel wall resistance post-AVF ligation. Alternatively, some suggest that arterial flow through AVF induces elevated wall stress and diminished wall thickness.

Brachial artery aneurysms typically manifest symptoms due to localized compression, with patients commonly experiencing arm pain, swelling, paresthesia, and fingertip discoloration [1].

Our patient exhibits a pulsatile throbbing mass at the elbow crease, accompanied by the absence of distal pulses, yet without signs of ischemia. We performed an aneurysm resection under an Esmarch bandage with an end-to-end anastomosis. The resection of an aneurysmal artery with end-to-end anastomosis is detailed in two reports [5,6]. This procedure is reserved for instances of limited aneurysm extension, as prolonged extension may result in increased wall tension. It is also considered when the diameter of the artery remains consistent.

In cases where the size of the aneurysm precludes a terminal anastomosis, the great saphenous vein can also serve as a graft. Several authors have reported excellent outcomes using the great saphenous vein as a graft [4,7-10].

Several authors have utilized polytetrafluoroethylene (PTFE) grafts and demonstrated that a prosthetic graft serves as a viable alternative when autologous veins are unavailable in their respective cases [4,6,7,11,12]. No complications were reported following the use of PTFE grafts.

No cases of endovascular treatment have been reported in the medical literature.

## Conclusions

This case report emphasizes the need for heightened clinical awareness of brachial artery aneurysm as a delayed complication following AVF ligation in hemodialysis patients. While rare, such aneurysms can pose serious risks if left untreated. Our patient benefited from early detection and timely surgical intervention, resulting in full recovery without complications. This case reinforces the importance of regular post-transplant vascular monitoring and individualized surgical planning. Future studies may explore the potential role of less invasive interventions, but open surgical repair remains the gold standard in selected cases.

## References

[1] Karatepe C, Yetim TD (2011). Treatment of aneurysms of hemodialysis access arteriovenous fistulas. Turk J Thorac Cardiovasc Surg.

[2] Gray RJ, Stone WM, Fowl RJ, Cherry KJ, Bower TC (1998). Management of true aneurysms distal to the axillary artery. J Vasc Surg.

[3] Sohrabi C, Mathew G, Maria N, Kerwan A, Franchi T, Agha RA (2023). The SCARE 2023 guideline: updating consensus Surgical CAse REport (SCARE) guidelines. Int J Surg.

[4] Teixeira S, Pinto PS, Veiga C, Silva I, Almeida R (2017). Aneurysmal degeneration of the brachial artery after vascular access creation: surgical treatment results. Int J Angiol.

[5] Chemla E, Nortley M, Morsy M (2010). Brachial artery aneurysms associated with arteriovenous access for hemodialysis. Semin Dial.

[6] Marconi M, Adami D (2015). Giant true brachial artery aneurysm after hemodialysis fistula closure. Eur J Vasc Endovasc Surg.

[7] Marzelle J, Gashi V, Nguyen HD, Mouton A, Becquemin JP, Bourquelot P (2012). Aneurysmal degeneration of the donor artery after vascular access. J Vasc Surg.

[8] Dammers R, Tordoir JH, Kooman JP, Welten RJ, Hameleers JM, Kitslaar PJ, Hoeks AP (2005). The effect of flow changes on the arterial system proximal to an arteriovenous fistula for hemodialysis. Ultrasound Med Biol.

[9] Keuter XH, Planken RN, van der Sande FM, Tordoir JH (2006). Brachial artery thrombosis due to haemodialysis arteriovenous fistula. Nephrol Dial Transplant.

[10] Eugster T, Wigger P, Bölter S, Bock A, Hodel K, Stierli P (2003). Brachial artery dilatation after arteriovenous fistulae in patients after renal transplantation: a 10-year follow-up with ultrasound scan. J Vasc Surg.

[11] Kordzadeh A, D'Espiney Barbara RM, Ahmad AS, Hanif MA, Panayiotopoulos YP (2015). Donor artery aneurysm formation following the ligation of haemodialysis arteriovenous fistula: a systematic review and case reports. J Vasc Access.

[12] Dinoto E, Bracale UM, Vitale G (2012). Late, giant brachial artery aneurysm following hemodialysis fistula ligation in a renal transplant patient: case report and literature review. Gen Thorac Cardiovasc Surg.

